# Methodology of health-related quality of life analysis in phase III advanced non-small-cell lung cancer clinical trials: a critical review

**DOI:** 10.1186/s12885-016-2152-1

**Published:** 2016-02-18

**Authors:** Frédéric Fiteni, Amélie Anota, Virginie Westeel, Franck Bonnetain

**Affiliations:** Methodology and Quality of Life in Oncology Unit, University Hospital of Besançon, Besançon, France; EA 3181 University of Franche-Comté, Besançon, France; The French National Platform Quality of Life and Cancer, Besançon, France; Department of Medical Oncology, University Hospital of Besançon, Besançon, France; Chest disease Department, University Hospital of Besançon, Besançon, France; EORTC QOL Group, Brussels, Belgium

**Keywords:** Health-related quality of life, Lung cancer, Methodology

## Abstract

**Background:**

Health-related quality of life (HRQoL) is recognized as a component endpoint for cancer therapy approvals. The aim of this review was to evaluate the methodology of HRQoL analysis and reporting in phase III clinical trials of first-line chemotherapy in advanced non-small cell lung cancers (NSCLC).

**Methods:**

A search in MEDLINE databases identified phase III clinical trials in first-line chemotherapy for advanced NSCLC, published between January 2008 to December 2014. Two authors independently extracted information using predefined data abstraction forms.

**Results:**

A total of 55 phase III advanced NSCLC trials were identified. HRQoL was declared as an endpoint in 27 studies (49 %). Among these 27 studies, The EORTC questionnaire Quality of Life Questionnaire C30 was used in 13 (48 %) of the studies and The Functional Assessment of Cancer Therapy-General was used in 12 (44 %) trials. The targeted dimensions of HRQoL, the minimal clinically important difference and the statistical approaches for dealing with missing data were clearly specified in 13 (48.1 %), 9 (33.3 %) and 5 (18.5 %) studies, respectively. The most frequent statistical methods for HRQoL analysis were: the mean change from baseline (33.3 %), the linear mixed model for repeated measures (22.2 %) and time to HRQoL score deterioration (18.5 %). For each targeted dimension, the results for each group, the estimated effect size and its precision were clearly reported in 4 studies (14.8 %), not clearly reported in 11 studies (40.7 %) and not reported at all in 12 studies (44.4 %).

**Conclusions:**

This review demonstrated the weakness and the heterogeneity of the measurement, analysis, and reporting of HRQoL in phase III advanced NSCLC trials. Precise and uniform recommendations are needed to compare HRQoL results across publications and to provide understandable messages for patients and clinicians.

## Background

The Food and Drug Administration (FDA) considers overall survival (OS) benefit as the foundation for the approval of new anticancer drugs in the United States [1]. Nevertheless, the increasing number of effective salvage treatments available in many types of cancer (i.e. subsequent lines of treatments) has resulted in the need for a larger number of patients to be included and/or the need of a more prolonged observation period to attain sufficient events that can achieve planned statistical power; this increases the cost of clinical trials and requires a longer duration to obtain results [2]. Consequently, intermediate endpoints such as progression-free survival are often used as primary endpoints because they are assessed earlier. However, there is a lack of consistency in their definitions [3] and they are not systematically validated as surrogate endpoints for OS.

In this context, HRQoL could constitute an alternative endpoint. HRQoL is recognized as a endpoint for cancer therapy approvals by the American Society of Clinical Oncology and the FDA [1, 4] HRQoL reflects the patient-perceived evaluation of one’s health, including physical, emotional, and social dimensions as well as symptoms due to disease or treatment. Several publications have underlined some key issues regarding the heterogeneity of HRQOL reporting in randomized clinical trials (RCTs) in oncology [5, 6]. Moreover, the statistical longitudinal analysis of HRQoL remains unstandardized which compromises the comparison of results between trials [7]. To improve the reporting of HRQoL in oncology randomized clinical trials, an extension to the CONSORT statement regarding HRQoL reporting was published. Nevertheless, these guidelines do not detail the methodology of statistical analysis of HRQoL. Claassens et al. reported that some aspects of HRQoL reporting (missing HRQOL data, a priori hypotheses of HRQOL, rationale for instruments used) remain underrepresented in non–small-cell lung cancer (NSCLC) RCTs [6].

The aim of this review was to evaluate the three major steps of HRQoL analysis: measurement, statistical analysis, results presentation in phase III clinical trials of first-line chemotherapy in advanced NSCLC with a special focus on the statistical analysis**.**

## Methods

### Search strategy and Selection for studies

Eligible trials were randomized phase III trials of first-line chemotherapy in advanced NSCLC. Literature searches in PubMed database (January 2008 to December 2014) were performed. Trials published from 2008 were included to assess HRQoL analysis in recent trials. For PubMed database research, the following strategies were used: (lung neoplasm[MeSH Terms]) AND (advanced[Text Word] OR metastatic[Text Word]) + filters Clinical Trial, Phase III. In case of companion papers (i.e. HRQoL analyses reported in a separate paper, not in the princeps one), only the information and methodology declared in the HRQoL paper were reported.

### Data extraction

Two authors (F. F., A.A) independently extracted information using predefined data abstraction forms. All data were checked for internal consistency, and disagreements were resolved by discussion among the investigators. The following details were extracted: general items (number of patients, year of publication, study period, number of centers, nationality of the first author, academic, mixed or industrial trial), name of the primary endpoint, items related to HRQoL measurement and reporting (rational for HRQoL assessment, methods of data collection, HRQoL questionnaire, evidence of HRQoL questionnaire validity, method/algorithm for scoring the questionnaire, planned schedule of questionnaires administration, results from each targeted dimensions for multidimensional questionnaires, method for results presentation, discussion of limitations and implications for generalizability and clinical practice.

We assessed statistical HRQoL analysis according to 13 key parameters: statement of the targeted dimensions, statement of the HRQoL hypothesis, procedure to control the type I error rate, statement of the minimal clinically important difference (MCID), data set for HRQOL analyses, description of the number of HRQoL data available at baseline and at subsequent time points, statement of HRQoL scores at baseline for each group and each dimension, profile of missing data at baseline, statement of statistical approaches for dealing with missing data, statistical approach for HRQoL analysis, MCID taken into account in the statistical analysis method and/or in the interpretation of the results, statement of the multivariate analysis. Each key point was coded as “yes” (2 points), “unclear” (1 point) or “no” (0 point). A score on a 0–26 scale was then created with a high score represent a high methodological level for statistical analysis.

### Data analyses

We conducted a descriptive analysis of selected publications, HRQoL measurement, reporting and of the statistical analysis of HRQoL with each key point.

Quantitative variables were descripted with median and range. Qualitative variables were descripted with absolute frequencies (number) and relative frequencies (proportion).

Analyses were conducted with the use of SAS software, version 9.3 (SAS Institute).

## Results

### HRQoL measurement

**Fig. 1:**
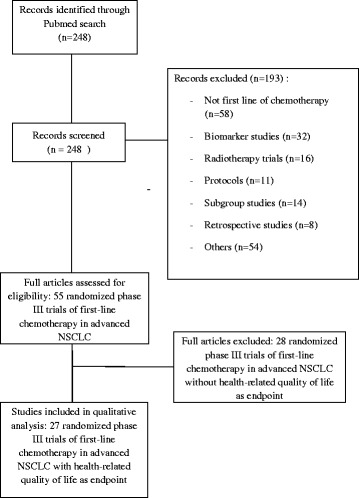
Identification randomized phase III trials of first-line chemotherapy in advanced non-small cell lung cancer (NSCLC) trials from PubMed search

A total of 55 phase III advanced NSCLC trials published between 2008 and 2014 were identified (Fig. 1). Among these studies, 27 trials were identified with HRQOL endpoint (49 %) (Fig. 1) including 2 studies with HRQOL as primary endpoint. Of the 27 studies, the background and rationale for HRQoL used was provided in 6 trials (22.2 %). For 5 trials (18.5 %), additional HRQoL publications were released in a separate HRQoL dedicated paper. Five trials (18.5 %) provided no result of HRQoL.

The EORTC Quality of Life Questionnaire C30 (QLQ-C30) was the most frequently used instrument. It was used in 13 (48 %) of the studies (Table 1). The lung cancer-specific module EORTC QLQ-LC13 was added to the QLQ-C30 in 12 studies (37 %). Among these 12 studies, two studies added the EuroQoL EQ-5D generic questionnaire to the QLQ-C30 and QLQ-LC13. The Functional Assessment of Cancer Therapy (FACT) questionnaires were used in 12 studies (44.4 %): one study (3.7 %) used the FACT-General (FACT-G), 7 studies (25.9 %) used the FACT-L questionnaire specific for lung cancer patients and 4 studies (14.8 %) used the FACT-LCS which is a subset of the FACT-L with 7 items (Table 1). The Lung Cancer Symptom Scale questionnaire was used in 1 study (3.7 %). One study did not specify the HRQoL questionnaire used. The reference of the HRQoL instrument validation was provided in 13 studies (48 %). Among the 4 studies using the FACT questionnaires in other than English version, none of them reported the transcultural validation. Six studies (22.2 %) defined the procedure of questionnaire completion (i.e. paper and pencil, electronic completion, at home or at the clinic). The planned schedule of HRQoL assessment was reported in 23 trials (85.2 %).

**Table 1:** Aspects relevant to HRQoL statistical analysis

First authors	HRQoL questionnaire	Timing of assessment	Targeted dimensions	MCID	Statistical approach for dealing with missing data	Statistical approach for HRQoL analysis
Wu [19]	QLQ-C30; QLQ-LC13	Randomization and every 3 weeks until disease progression or new cancer treatment	Cough, dyspnoea, pain	10 points		Distribution of patients whose symptom had improved, remained stable, or worsened; the time to deterioration of symptoms; mixed-effects growth curve model
Laurie [20]	QLQ-C30; QLQ-LC13 and two additional questions (hand-foot syndrome and headache)	At baseline and every cycle				
Yang [7]	QLQ-C30; QLQ-LC13	Random assignment and every 21 days until disease progression	Cough, dyspnoea, pain	10	Joint analysis	Time to deterioration; mixed-effects growth curve model
Wu [19]	FACT-L	Not available	FACT-L total score and TOI			Time to deterioration
Yoshioka [21]	FACT-L; FACT/GOGNTX	At the time of enrollment and at 6 and 9 weeks after the initiation of treatment				Linear mixed model for repeated measures
Lee [22]	QLQ-C30, QLQ-LC14, EUROQOL	Baseline, monthly during the first year, then 18 and 24 months after randomization				Linear mixed model for repeated measures
Gridelli [23]						
Flotten [24]	QLQ-C30, QLQ-LC13	Before each cycle, 3 weeks after the last cycle, and then every 8 weeks until 57 weeks	Global health status health, nausea/vomiting, dyspnoea, fatigue	10		Mean change from baseline
Socinsky [25]	FACT-G, FACTTAXANE	Baseline and on day 1 of each cycle	Peripheral neuropathy, pain, hearing, edema			Mean change from baseline
Groen [26]	QLQ-C30, QLQ-LC13	Start of chemotherapy and weeks 6,12,16,24,30				Mean change from baseline
Chen [9]	FACT-L	Baseline and every 6 weeks		6 for FACT-L total score and TOI; 2 points for LCS LCS		Mean change from baseline
Lara [27]	QLQ-C30	On day 1 of each odd cycle and at the end of the treatment visit				Mean change from baseline
Koch [12]	QLQ-C30, QLQ-LC13	Baseline, weeks 3,6,9,12,20,28	Dyspnoea, fatigue, pain, pain medication, global health status			Group comparisons of scale at each time; mean change from baseline; AUC; rates of symptom palliation
Biesma [28]	QLQ-C30, QLQ-LC13	Before, during (day 1 and day 8 of each cycle) and after each cycle (weeks 12,15,18)	Global health status	10		Mean change from baseline and week 18; linear mixed model for repeated measures
Weissman [29]	FACT-L	After each cycle	TOI			Mean change from baseline and after 6 cycles
Okamoto [30]	FACT-L, FACT/GOGNTX	Time of enrollment and at 6 and 9 weeks after initiation of treatment				Linear mixed model for repeated measures
Thongpraset [31]	FACT-L	Baseline, weeks 1 AND 3, 3-weekly until week 18, 6 weekly until progression and at discontinuation	FACT-L Score, TOI, LCS	6 for FACT-L total score and TOI; 2 points for LCS	If less than 50% of the Fact-l subscale scores were missing, the subscale score was divided by the number of completed items and multiplied by the total number of items on the scale. If 50% or more of the items were missing, that subscale was treated as missing for that patient	Mean change from baseline; Time to worsening; Time to improvement
Lynch [32]	FACT-L	Baseline, before each treatment cycle and at the end of the therapy				Mean change from baseline; Time to symptomatic disease progression
Takeda [33]	FACT-L	The time of enrollment and at 12 weeks, 18 weeks after initiation of treatment	LCS		Linear mixed-effects model in which the missing data depend on the observed score	Linear mixed model for repeated measures
Lee [34]	QLQ-C30, QLQ-LC14	Random, During each cycle, at the end of the chemotherapy, every 6 months until 24 months				Linear mixed model for repeated measures
Treat [35]	FACT-L	Not available				
Tan [36]	Lung cancer symptom scale	Baseline, at the end of each cycle, just before the next cycle, at the end of the study				
Pirker [37]	QLQ-C30, QLQ-LC13, EuroQoL EQ-5D	Not available				
Gronberg [38]	QLQ-C30, QLQ-LC13	Weeks 0,3,6,9,12,20,28,36,44,52	Global health status health, nausea/vomiting, dyspnoea, fatigue	10	Last value carried forward for missing value that followed, even after death	Area under the curve
O’Brien [39]	FACT-L	Baseline, before each cycle and at the end of the treatment	LCS	2	Missing scores at week 3 were classified as having less than a 2-point increase in the primary analysis data but classified as missing and excluded from the supplemental analysis	Fisher test for equal proportion of patients achieving at least two points increase
Langer [40]	FACT-L	Baseline and within 3 days of each treatment	LCS	2		Percentage of patients with at least two points improvement at the beginning of cycle 2
Gebbia [41]	QLQ-C30; QLQ-LC13	Baseline and every cycle				

### Statistical analysis of HRQoL

The Table 2 summarized the quality of statistical analysis of HRQoL.

**Table 2:** The 13 keys parameters for statistical HRQoL analysis assessed as “yes” if the authors specified the parameter, “not clear” it was not clear and “no” if the authors didn’t specify the parameter

	Yes, n (%)	Not clear, n (%)	No, n (%)
Targeted dimensions	13 (48.1)	0	14 (51.9)
HRQoL hypothesis	2 (7.4)	0	25 (92.6)
Procedure to control the type I error	1 (3.7)	1 (3.7)	25 (92.6)
Minimal clinically important difference	9 (33.3)	1 (3.7)	17 (63)
Study population	3 (11.1)	3 (11.1)	21 (77.8)
Number of HRQoL data at subsequent time points	7 (25.9)	6 (22.2)	14 (51.9)
HRQoL scores at baseline for each group and each dimension	6 (22.2)	2 (7.4)	19 (70.4)
Profile of missing data at baseline	1 (3.7)	2 (7.4)	24 (88.9)
Statistical approaches for dealing with missing data	5 (18.5)	0	22 (81.5)
Statistical approach for HRQoL analysis	14 (51.9)	1 (3.7)	12 (44.4)
MCID taken into account in the statistical analysis	7 (25.9)	2 (7.4)	18 (66.7)
Multivariate analysis	1 (3.7)	0	26 (96.4)

The mean score based on the 13 key parameters of the 27 trials was 6.3 (standard deviation = 6.1, range = 0-20).

The targeted dimensions of HRQoL were pre-specified in 13 studies (48.1 %) in the method section: 6 of them used EORTC questionnaires and 7 used FACT questionnaires. Among the 6 studies which used EORTC questionnaires, 3 dimensions were targeted in mean and targeted dimensions were dyspnea (66.6 %), global health status (66.6 %), pain (50.0 %), fatigue (50.0 %), cough (33.3 %) and nausea/vomiting (33.3 %) (Table 1). Among the 7 studies which used FACT questionnaires, 2 dimensions were targeted in mean and most frequent targeted dimensions were the lung cancer subscale (57.2 %), the trial outcome index (57.2 %) and the FACT-L global score (28.6 %) (Table 1).

Nine studies (33.3 %) defined the MCID. Among these studies: 5 used the EORTC questionnaires and all of them used a 10-point decrease in the HRQoL scores as the MCID (Table 1), 4 used the FACT questionnaires and all of them used 6 points decrease for the FACT-L global score and trial outcome index, and 2 points decrease for the lung cancer subscale as the MCID.

The MCID was taken into account in the statistical analysis and/or in the interpretation of the results in 7 studies (25.9 %) (Table 1).

Only three studies mentioned the population data set for HRQoL analysis: 2 in modified intention-to-treat, 1 in intention-to-treat. Definition for the mITT population were 1) “all randomly assigned patients with data were included” [8] and 2) “patients with a baseline and at least one post-baseline HRQoL assessment were included” [9].

The number of HRQoL data at baseline and at subsequent time points, the HRQoL scores at baseline for each group and each dimension, the profile of missing data at baseline, the statistical approaches for dealing with missing data were adequately reported in 7 (25.9 %), 6 (22.2 %), 1 (3.7 %) and 5 (18.5 %) studies, respectively (Table 2). The statistical methods for dealing with missing data were different (Table 1). No study provided the reasons why data were missing.

Fourteen studies (51.9 %) described the statistical approach to analyze HRQoL data in the method section (Table 2). Seven studies used two or more statistical approaches. The different statistical methods/analyses were: the mean change from baseline (33.3 %), the linear mixed model for repeated measures (LMM) (22.2 %), time to HRQoL score deterioration (TTD) (18.5 %), AUC (7.4 %), mixed-effects growth-curve model (7.4 %), distribution of patients whose symptom had improved, remained stable or worsened (3.4 %), Fisher test for equal proportion of patients achieving at least two points increase (3.4 %), group comparisons of scale at each time (3.4 %), rates of symptom palliation (3.4 %), percentage of patients with at least two points improvement at the beginning of the cycle two (3.4 %) (Table 1). No study specified which were the primary statistical analysis and the sensitive analysis.

Among the six studies which used a LMM to analyze the longitudinal HRQoL data, none of them declared the effects introduced in the model (random or fixed effects and interactions).

One study presented a multivariate analysis (Table 2).

### HRQoL results presentation

For each targeted dimension, the results for each group, the estimated effect size and its precision were adequately reported in four studies (14.8 %), not clearly reported in 11 studies (40.7 %) and not reported at all in 12 studies (44.4 %). A discussion specific limitations and implications for clinical practice was provided in 10 articles (37 %) (Table 1).

The results were presented in the text, figures or tables in 24 (88.9 %), 12 (44.4 %) and 8 (29.6 %) articles, respectively (Table 1).

## Discussion

In this review, we showed that HRQoL was declared as an endpoint in only 49 % of the phase III clinical trials in advanced NSCLC published between 2008 and 2014. Moreover, we clearly demonstrated the heterogeneity and the weakness of the methodology of HRQoL measurement, statistical analysis and reporting. First, two questionnaires are the most widely used: the QLQ-C30 plus LC13 module (48 %) and the FACT-L (44 %). As an example, the OPTIMAL [8, 9] and the Lux-Lung 3 [10] trials compared the efficacy and tolerability of two tyrosine kinase inhibitor (erlotinib and afatinib, respectively) versus chemotherapy in first-line line treatment of patients with advanced EGFR mutation-positive NSCLC. HRQoL was assessed by the FACT-L questionnaire in OPTIMAL trial [9] while EORTC QLQ-C30 and LC13 module were used in LUX-Lung 3 trial [7]. These two clinical trials could hardly be compared since EORTC and FACT questionnaires for lung cancer do not contain the same dimensions in regard to impact of lung cancer on HRQoL. Moreover, two studies used the EuroQoL EQ-5D generic questionnaire which comprises only five short questions and is suitable since it limits patient burden and in that way also encourages response rate. Nevertheless this questionnaire has not been tailored to the special requirements of patients with cancer. At this time, the foremost challenge would be to promote, through cancer site and treatment modalities, guidelines for selecting the best questionnaires allowing for direct comparison of results across trials. Moreover, it is also still necessary to develop some new tools to evaluate HRQoL. Already validated questionnaires may not be adapted to new targeted biotherapy agent which can induce some long-term moderate toxicities.

Then, the number of HRQoL measures and intervals between two consecutive measures vary from one study to another. HRQoL is often captured until tumor progression, nevertheless, in advanced NSCLC, we could wonder if it would be more appropriate to measure HRQoL until death. Recommendations on the schedule of HRQoL assessment should be provided. At least three HRQoL assessment are recommended: at baseline, during treatment and at the end of the study (http://groups.eortc.be/qol/eortc-qlq-c30). However, a more intensive HRQoL assessment is preferable to better capture the longitudinal trajectory of HRQoL level and to capture any relevant changes. In this review, most of studies evaluate HRQoL at baseline and then every treatment cycle which allow a good appreciation of the impact of treatment of HRQoL over time, but it depends on the number of cycles received by the patient. Moreover attention should be paid to the timing of diagnostic procedures which could influenced HRQoL results, especially with lifethreatening cancers. Patients are likely to be experiencing stress in anticipation of the yet unknown results. After the procedure, the patients will either be experiencing great relief or anxiety depending on the results. This point should be taken into account in HRQoL assessment design and be carefully documented in the protocol and emphasized during training.

The *a priori* selection of the targeted dimensions was heterogeneous between the trials and was pre-specified only 13 times (48.1 %) in the method section and most of them are symptomatic scales. We know that there is general agreement concerning the multidimensional concept of HRQoL taking into account levels of physical, mental, social, and patient satisfaction with treatment. Therefore, the choice of only symptomatic HRQoL dimensions reaches the problem of the holistic sense of HRQoL. Moreover, the choice of the targeted dimensions of HRQoL must be discussed between clinicians and methodologists and clearly described in the protocol.

In confirmatory clinical trials with multiple endpoints, the use of multiple test procedures is mandatory and CONSORT Statement recommends a multiplicity adjustment in case of multiple testing [11]. However, only one study clearly stated the procedure to control the type I error [12].

Prior to longitudinal HRQoL data analysis, the MCID should be a priori determined [13]. In our review, only nine studies (33.3 %) clearly specified it. The MCID represents the smallest changes/differences in HRQoL score, which is perceived as clinically important. For the EORTC questionnaires, a 5-point to a 10-point difference in scores could be considered as the MCID [14]. In patients with NSCLC, Maringwa et al. [15] tried to determine the smallest changes in HRQOL scores in a subset of the EORTC QLQ-C30 scales, which could be considered as clinically meaningful. They concluded that the estimates of 5 to 10 units of the QLQ-C30 scales may be used as guidance for clinicians and researchers to classify patients as improved or deteriorated. In our review, the 5 studies which used the QLQ-C30 and stated the MCID, all used a 10-point decrease in the HRQoL scores as the MCID. A sensitivity analysis, with a MCID of 5 points could have been proposed to assess the robustness of the results.

Missing data, considered as missing not at random, can bias the longitudinal analysis if it is not adequately taken into account [16]. Patients may drop out before the planned end of the study, resulting in the absence of any available HRQoL data after the patient’s drop out (i.e. attrition). Moreover, drop out occurs generally due to a deterioration of patient health status or death. Patients may also be too tired to fill the questionnaire entirely at a specific measurement time. This induces the potential risk to select subpopulation of patients with better HRQoL levels and with available HRQoL data. Not adjusting for missing data can limit the robustness of the results and the confidence in the HRQoL conclusions. Therefore, the profile of missing data at baseline and the number of HRQoL at subsequent time points for each group must be specified. In our review, these two information were reported in only 1 (3.7 %) and 7 (25.9 %) trials, respectively. Furthermore, only 5 studies (18.5 %) described the statistical approaches for dealing with missing data and the 5 methods were different. Thus, the reporting of missing data and the statistical approaches of analysis of missing data need to be standardized.

There are a number of possible ways of analyzing longitudinal HRQoL data. Currently, the two main robust methods are: the LMM and the TTD [6, 17]. In our review, the most widely used is the mean change from baseline (33.3 %). This method summarizes the longitudinal data into a summary statistic before performing a between-arms comparison. This method is rather simple but may overlook important changes in HRQoL along time and should not be applied. This method is not a model of longitudinal analysis. The LMM method was used in 6 studies (22.2 %). In all these studies the fixed effects, the random effects and the correlation matrix between HRQoL measures introduced in the model were not specified. Thus, results are very difficult to interpret. The LMM can estimate a time effect, an arm effect and an interaction between treatment arm and time (reflecting a different evolution of the two treatment arms over time). The LMM contains both fixed effects (reflecting average trends) such as treatment and random effects (individual trends). This model accounts for the association (i.e. correlation) of measures made on the same patient at different times.

Finally, the LMM model generally require a normally distribution of the score studied. All studies using this method did not mention this hypothesis and a priori did not check it. For EORTC questionnaires, scores generally do not respect a normal distribution due to the low number of items per dimension. The TTD approach was used in 5 studies (18.5 %). Currently the definition of the TTD is not standardized; therefore the studies must clearly define it. The definition of the TTD must include: reference score, the event of interest, the censoring process, including death or not [6]. In our review, only one study defined the TTD: “Time to deterioration in patient-reported outcomes was measured in months from random assignment to the first instance of symptom worsening (10 points from baseline). Patients without worsening, including those with disease progression, were censored at the last available patient-reported outcome assessment; those lacking post-baseline assessments were censored at random assignment. Patients who died without documented worsening were considered to have deteriorated at the time of death” [7]. Other statistical methods were used: “distribution of patients whose symptom had improved, remained stable or worsened”, “Fisher test for equal proportion of patients achieving at least two points increase”, “group comparisons of scale at each time”, “rates of symptom palliation”, “percentage of patients with at least two points improvement at the beginning of the cycle two”. These methods can guide on the choice of the most appropriate analytical method to use to analyze longitudinal HRQoL data (e.g., the LMM or the TTD) approach. However, these analyses alone cannot be sufficient to capture all information present in the longitudinal HRQoL assessment and must be completed by a longitudinal statistical approach taking into account the correlation between HRQoL measures.

Finally, it should be acknowledged that journal space is often limited, and authors may not have been able to report all the methodology. Therefore, HRQoL may systematically be reported in separate HRQoL dedicated manuscripts.

## Conclusions

Our review demonstrated the poor quality and the heterogeneity of the measurement, analysis, and reporting of HRQoL in phase III advanced NSCLC trials. The heterogeneity between trials limits their cross comparison and the feasibility of meta-analysis.

The HRQoL CONSORT statement regarding HRQoL reporting was published in 2013 [18] and it is true that we reviewed studies published before 2013. The use of the HRQoL CONSORT extensions should be encouraged. Nevertheless, these guidelines do not detail the methodology of statistical analysis of HRQoL. Incomplete or inaccurate statistical analysis of HRQoL can affect the reliability of these outcomes. Therefore, development of guidelines for longitudinal HRQoL analysis of clinical trials is important to facilitate interpretation of HRQoL findings.

## Abbreviations

HRQoL: Health-related quality of life
NSCLC: Non-small cell lung cancer
FDA: Food and Drug Administration
OS: Overall survival
RCT: Randomized clinical trial
MCID: Minimal clinically important difference
EORTC QLQ-C30: European organization for research and treatment of cancer quality of life questionnaire C30
FACT: Functional Assessment of Cancer Therapy
LMM: Linear mixed model for repeated measures
TTD: Time to health-related quality of life score deterioration
